# Impact of cardiac history and myocardial scar on increase of myocardial perfusion after revascularization

**DOI:** 10.1007/s00259-023-06356-4

**Published:** 2023-08-10

**Authors:** Ruurt A. Jukema, Ruben W. de Winter, Luuk H.G.A. Hopman, Roel S. Driessen, Pepijn A. van Diemen, Yolande Appelman, Jos W.R. Twisk, R. Nils Planken, Pieter G. Raijmakers, Paul Knaapen, Ibrahim Danad

**Affiliations:** 1 Departments of Cardiology, Amsterdam Cardiovascular Sciences, Amsterdam UMC, Vrije Universiteit Amsterdam, Amsterdam, the Netherlands; 2 Epidemiology & Data Science, Amsterdam UMC, Vrije Universiteit Amsterdam, Amsterdam, the Netherlands; 3 Radiology, Nuclear Medicine & PET Research, Amsterdam UMC, Vrije Universiteit Amsterdam, Amsterdam, the Netherlands; 4 Department of Cardiology, University Medical Center Utrecht, Heidelberglaan 100, 3584 CX Utrecht, The Netherlands

**Keywords:** Revascularization, PCI, CABG, Perfusion, FFR

## Abstract

**Purpose:**

We sought to assess the impact of coronary revascularization on myocardial perfusion and fractional flow reserve (FFR) in patients without a cardiac history, with prior myocardial infarction (MI) or non-MI percutaneous coronary intervention (PCI). Furthermore, we studied the impact of scar tissue.

**Methods:**

Symptomatic patients underwent [^15^O]H_2_O positron emission tomography (PET) and FFR before and after revascularization. Patients with prior CAD, defined as prior MI or PCI, underwent scar quantification by magnetic resonance imaging late gadolinium enhancement.

**Results:**

Among 137 patients (87% male, age 62.2 ± 9.5 years) 84 (61%) had a prior MI or PCI. The increase in FFR and hyperemic myocardial blood flow (hMBF) was less in patients with prior MI or non-MI PCI compared to those without a cardiac history (FFR: 0.23 ± 0.14 vs. 0.20 ± 0.12 vs. 0.31 ± 0.18, *p* = 0.02; hMBF: 0.54 ± 0.75 vs. 0.62 ± 0.97 vs. 0.91 ± 0.96 ml/min/g, *p* = 0.04). Post-revascularization FFR and hMBF were similar across patients without a cardiac history or with prior MI or non-MI PCI. An increase in FFR was strongly associated to hMBF increase in patients without a cardiac history or with prior MI/non-MI PCI (*r* = 0.60 and *r* = 0.60, *p* < 0.01 for both). Similar results were found for coronary flow reserve. In patients with prior MI scar was negatively correlated to hMBF increase and independently predictive of an attenuated CFR increase.

**Conclusions:**

Post revascularization FFR and perfusion were similar among patients without a cardiac history, with prior MI or non-MI PCI. In patients with prior MI scar burden was associated to an attenuated perfusion increase.

**Supplementary Information:**

The online version contains supplementary material available at 10.1007/s00259-023-06356-4.

## Introduction

Current revascularization strategies for patients with chronic coronary syndrome are aimed at detecting ischemia-causing coronary stenoses, restoration of myocardial perfusion by percutaneous coronary intervention (PCI) or coronary artery bypass grafting (CABG) and, subsequently, relief of symptoms [1]. To maximize the effect of revascularization therapy, guidelines recommend to assess the presence of inducible ischemia beforehand [1]. Fractional flow reserve (FFR) is an important vessel-specific tool to assess myocardial ischaemia and since post-procedural FFR and myocardial blood flow (MBF) have been linked to superior outcomes, the use of FFR and MBF has been extended to a post-revascularization tool [2, 3]. A previous study by Driessen et al. included patients without prior myocardial infarction (MI) or PCI and showed that an increase in FFR was paralleled by improvement of hyperemic MBF with a strong correlation between these two indices (*r* = 0.74) [4]. However, the relationship between FFR and absolute myocardial perfusion is not only determined by epicardial coronary stenoses, but also by microvascular resistance and the subtended myocardial mass. As such, a supposedly increase in FFR is not necessarily commensurate with an equivalent increase in hyperemic blood flow (hMBF). Microvascular disease is encountered in up to 50% of patients with prior infarction and the revascularization benefit in terms of perfusion increase and as such symptom reduction has been questioned in this group of patients [5]. Remarkably, data on the restoration of myocardial blood flow after revascularization therapy in patients with prior MI or PCI is lacking. As such, we investigated the influence of revascularization on FFR and absolute myocardial perfusion in patients without a cardiac history, with prior MI or non-MI PCI using serial FFR and positron emission tomography (PET) MBF measurements. Furthermore, we investigated whether the restoration of myocardial perfusion is attenuated by scar tissue burden or conventional risk factors.

## Methods

### Patient selection

This is a sub study of the Comparison of Coronary CT Angiography, SPECT, PET, and Hybrid Imaging for Diagnosis of Ischemic Heart Disease Determined by Fractional Flow Reserve (PACIFIC 1) and Functional stress imaging to predict abnormal coronary fractional flow reserve: the PACIFIC 2 study (PACIFIC 2), which were prospective clinical single-centre, head-to-head comparative studies conducted from 2012 to 2020, at the Amsterdam UMC, VU University Medical Centers, Amsterdam, the Netherlands [6, 7]. All patients were suspected of having stable obstructive coronary artery disease (CAD), were referred for a clinically indicated diagnostic ICA, and underwent a 2-week protocol in which patients underwent [^15^O]H_2_O PET prior to invasive coronary angiography (ICA) with routine 3 vessel invasive FFR interrogation. Patients suspected for acute coronary syndrome were not included. Additionally, patients with a cardiac history, defined as prior MI or PCI, underwent cardiac magnetic resonance (CMR) imaging with late gadolinium enhancement (LGE) prior to invasive coronary angiography (ICA). This PACIFIC post-hoc analysis included patients in whom FFR interrogation or [^15^O]H_2_O PET perfusion imaging was repeated after coronary revascularization (PCI or CABG). Patients with events between revascularization and follow-up PET (*n* = 1) were excluded. The study complied with the Declaration of Helsinki. The study protocol was approved by the VUmc Medical Ethics Review Committee and all patients provided written informed consent.

### PET

The PET scans were performed on a hybrid PET/CT device (Philips Gemini TF 64 or Ingenuity TF 128, Philips Healthcare, Best, The Netherlands). A 6-min dynamic scan protocol commencing simultaneously with an injection of 370 MBq [^15^O]H_2_O during resting and adenosine (140 µg/kg/min) induced hyperemic conditions. The dynamic scan sequence was followed by a low-dose CT-scan for attenuation correction. Parametric images of quantitative hyperemic MBF in ml/min/g were generated by in-house developed software (CardiacVUer, Amsterdam UMC, Vrije Universiteit Amsterdam, The Netherlands) [8] for each of the 17 left ventricle segments according to the standard American Heart Association model with standardized allocation of segments to the three vascular territories [9]. Patients were instructed to refrain from the intake of xanthine or caffeine 24 h prior to the PET. Parametric MBF images were analyzed. Regional hMBF was defined as mean hMBF of the entire vascular territory in the absence of a perfusion defect or as the mean hMBF of the perfusion defect (≥ 2 adjacent segments with a hMBF ≤ 2.3 ml/min/g) when present [10]. In the presence of an inferior perfusion defect the invasive angiography was used to determine coronary dominance and the perfusion defect was allocated accordingly. Regional hMBF of the predefined vascular territories was used for analysis.

### CMR

Images were acquired on a 1.5-T whole body MR scanner (Magnetom Avanto, Siemens Healthineers). Left ventricular (LV) cardiac function was assessed in between stress and rest perfusion with steady-state free-precession cine imaging in the 2-, 3-, 4-chamber long-axis views and multiple short-axis views covering the LV from base to apex. Late gadolinium enhancement (LGE) was performed using a 2-dimensional segmented inversion-recovery gradient-echo pulse sequence. If LGE was considered visually present, total and segmental infarct size (in grams) was calculated from the LGE images using the full width at half maximum method [11]. Infarct mass was also expressed as a percentage of myocardial mass per segment according to the AHA 17-segment model excluding the apex [9]. Additionally, in accordance with the AHA model, infarct size and percentage for each vascular territory was calculated. The segments used for PET were also used for scar analysis. LGE analysis was performed using Circle CVI42 (version 5.13, Circle Cardiovascular Imaging, Inc, Calgary, Canada) by a researcher blinded to clinical characteristics.

### ICA and FFR

ICA was performed according to standard clinical protocols [6]. Patients were instructed to refrain from the intake of xanthine or caffeine 24 h prior to the ICA. All major coronary arteries were routinely interrogated by FFR irrespective of stenosis severity and imaging results, except for occluded vessels or subtotal lesions with a diameter stenosis (DS) ≥ 90%. To induce maximal coronary hyperemia, adenosine was administered intracoronary as a 150 μg bolus. FFR was calculated as the ratio of mean distal intracoronary to aortic guiding pressure during hyperemia. The type of treatment (PCI, CABG or conservative) was left to the discretion of the operator and the heart team after consideration of symptoms, FFR and angiographic results. In case of PCI, post-procedural FFR was measured at the same location as the pre-PCI FFR. To evaluate the extent and diffuseness of atherosclerotic disease segment involvement scores were calculated according to Min et al., which is the sum of the number of segments with plaque irrespective of the degree of luminal stenosis [12].

### Statistical analysis

Continuous variables are expressed as mean ± SD or median (interquartile range) where appropriate. Categorical variables are presented as frequencies with percentages. Baseline characteristics between two groups were compared by the independent sample's T-test for continuous variables and the chi-square test for categorical variables. The correlation between two variables (FFR, hMBF, CFR or scar) was analyzed using Pearson’s correlation analysis. Paired FFR and regional perfusion measurements (before and after revascularization) were compared by the paired samples T-test. Regional perfusion and FFR analyses were stratified for patients without prior CAD, with prior MI or with a non-MI PCI. Patients with both a prior MI and with a non-MI PCI were grouped as prior MI. The change in FFR and perfusion was compared between these groups using an one way analysis of variance (ANOVA). In case the overall F-test for the ANOVA was significant, posthoc pairwise comparisons between patient categories were performed with Bonferroni correction for multiple comparisons. To analyze the importance of baseline FFR a sensitivity analysis stratified for vessels with FFR greater or less than 0.75 was performed. To identify predictors of regional perfusion improvement an analyses with regionally matched scar was performed using a mixed models with a random effect for subjects. Significant (*p* < 0.15) variables in the univariable analysis were included in the multivariable model. A two-sided P value < 0.05 was considered statistically significant. All statistical analyses were performed using IBM SPSS software package version 26 (IBM SPSS Statistics, IBM Corporation, Armonk, NY).

## Results

The study population consisted of 137 patients with 200 revascularized vessels. The mean age was 62.2 ± 9.5 years and 119 were male (86.9%). A total of 84 patients (61.3%) had a history of myocardial infarction or PCI. Patients were revascularized by PCI (*n* = 116, 84.7%) or CABG (*n* = 21, 15.3%). Further patient baseline characteristics are shown in Table 1. Vessel specific characteristics are shown in Table 2. In general, patients with prior MI or non-MI PCI had a more extensive cardiovascular risk profile. The median interval between revascularization and post revascularization PET was 34 days (interquartile range 21 to 58 days). Detailed flow charts describing PET and FFR availability are shown in supplemental Figs. 1, 2 and 3. Serial FFR measurements were available in 94 (47%) of the revascularized vessels, whereas paired hMBF and CFR measurements were available in 189 (95%) and 184 (92%) of the revascularized myocardial territories (supplemental Fig. 3). Baseline and post revascularization regional perfusion indices and FFR (including rest MBF) are depicted in Table 3 and supplemental Table 1.

**Table 1:** Baseline characteristics

	All patients *n* = 137	No cardiac history *n* = 53	Prior MI *n* = 41	Prior non-MI PCI *n* = 43	*P* value
Characteristics					
Male	119 (87%)	46 (87%)	36 (88%)	37 (86%)	0.97
Age, years	62.2 ± 9.5	58.7 ± 9.0	63.4 ± 8.3	65.5 ± 9.8	< 0.01
BMI, kg/m^2^	27.0 ± 3.9	27.1 ± 3.9	26.5 ± 3.5	27.4 ± 4.4	0.56
LVEF (%)	n/a	Estimated > 50%	56.6 (9.7)	59.9 (9.2)	n/a
Cardiovascular risk factors					
Diabetes Mellitus	29 (21%)	7 (13%)	12 (29%)	10 (23%)	0.19
Hypertension	76 (56%)	21 (40.0%)	27 (66%)	28 (65%)	0.01
Hypercholesterolemia	88 (64%)	25 (47%)	33 (81%)	30 (70%)	< 0.01
History of smoking	70 (51%)	29 (54%)	21 (51%)	20 (47%)	0.73
Family history of CAD	66 (48%)	26 (49%)	20 (49%)	20 (47%)	0.97
Medication					
Antiplatelet therapy	135 (99%)	51 (96%)	41 (100%)	43 (100%)	0.20
Β-blocker	90 (66%)	41 (77%)	24 (59%)	25 (58%)	0.07
Calcium channel blocker	47 (34%)	16 (30%)	12 (29%)	19 (44%)	0.26
ACE-inhibitor	38 (28%)	12 (23%)	12 (29%)	14 (33%)	0.54
ARB	27 (20%)	6 (11%)	11 (27%)	10 (23%)	0.14
Statin	112 (82.0%)	42 (80%)	35 (85%)	35 (81%)	0.75
Long acting nitrate	25 (18%)	5 (9%)	8 (20%)	12 (28%)	0.06
Symptoms					
Typical AP	67 (49%)	23 (43%)	21 (51%)	23 (54%)	0.56
Atypical AP	24 (18%)	13 (25%)	6 (15%)	5 (11%)	0.22
Non-specific chest discomfort	46 (34%)	17 (32%)	14 (34%)	15 (35%)	0.96

Mean ± SD, median (inter-quartile range) or N(%)

*AP* angina pectoris; *ARB* angiotensin II receptor blocker; *BMI* body mass index; *CAD* coronary artery disease; *LVEF* left ventricle ejection fraction; *MI* myocardial infarction; *PCI* percutaneous coronary intervention

**Table 2:** Depicts mean ± SD or median (inter-quartile range) for vessel-specific characteristics. Median (inter-quartile) infarct size (LGE) was only given for revascularized territories

Characteristics Total patients	All patients *n* = 137	No cardiac history *n* = 53	Prior MI *n* = 41	Prior non-MI PCI *n* = 43	*P* value
All vessels	*n* = 411	*n* = 159	*N* = 123	*N* = 129	
Revascularized	200 (49%)	90 (57%)	54 (44%)	56 (43%)	0.04
PCI	149 (75%)	47 (52%)	46 (85%)	56 (100%)	< 0.01
CABG	51 (26%)	43 (48%)	8 (15%)	0 (0%)	< 0.01
LGE (% of territory, *n* = 106)	n/a	n/a	0.6 (0.0–13.0_	0.0 (0.0–0.63)	n/a
Per vessel					
LAD					
Revascularized	93	44 (83%)	26 (63%)	23 (53%)	< 0.01
PCI	72 (78%)	26 (59%)	23 (89%)	23 (100%)	< 0.01
CABG	21 (23%)	18 (41%)	3 (12%)	0 (0%)	< 0.01
LGE (% of territory, *n* = 44)	n/a	n/a	0.0(0.0 –10.7)	0.0 (0.0–0.1)	
RCX					
Revascularized	56	23 (43%)	12 (29%)	21 (49%)	0.17
PCI	40 (71%)	10 (44%)	9 (75%)	21 (100%)	< 0.01
CABG	16 (29%)	13 (57%)	3 (25%)	0 (0%)	< 0.01
LGE (% of territory, *n* = 31)	n/a	n/a	4.4 (0.2 – 27.8)	0.0 (0.0–3.1)	n/a
RCA					
Revascularized	51	23 (43%)	16 (39%	12 (28%)	0.28
PCI	37 (73%)	11 (48%)	14 (88%)	12 (100%)	< 0.01
CABG	14 (28.5%)	12 (52%)	2 (13%)	0 (0%)	< 0.01
LGE (% of territory, *n* = 28)	n/a	n/a	1.0 (0.0–14.4)	0.0 (0.0–1.4)	n/a

*CABG* coronary artery bypass; *CAD* coronary artery disease; *LGE* late gadolinium enhancement, *LV* left ventricle

**Fig. 1:**
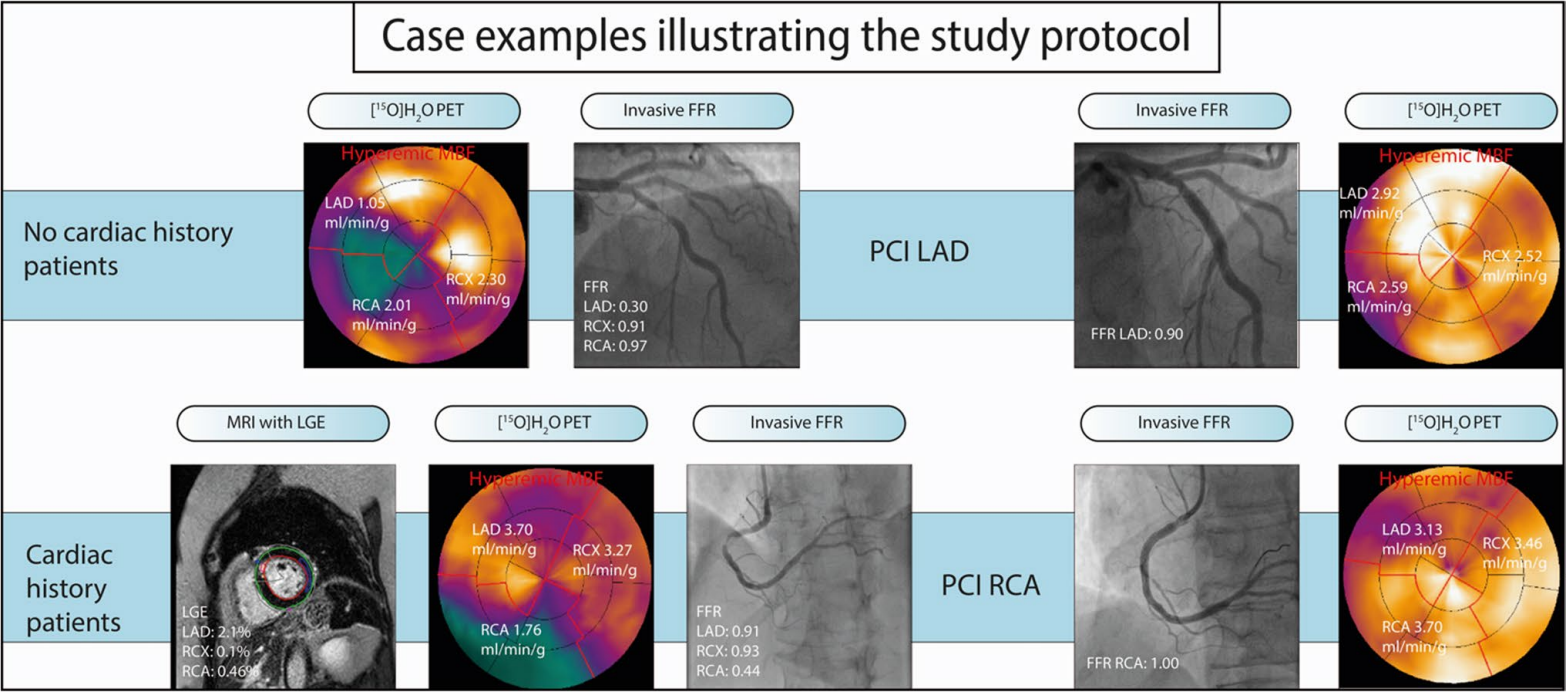
Case examples showing the effect of revascularization on FFR and perfusion in patients with and without a prior cardiac history. Abbreviations: FFR, fractional flow reserve; LAD, left anterior descending artery; LGE, late gadolinium enhancement; PET, positron emission tomography; RCA, right coronary artery

**Fig. 2:**
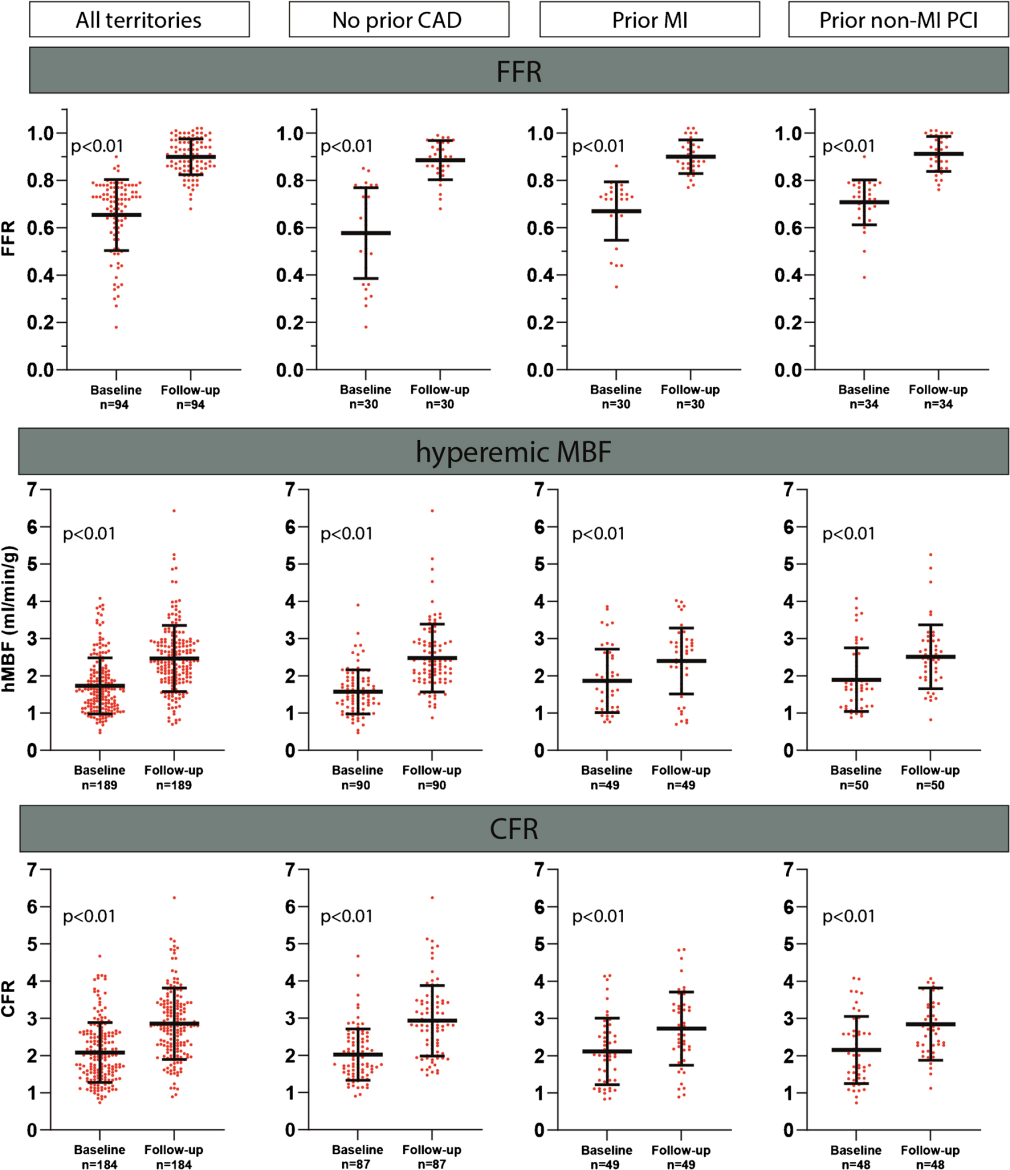
Change of regional perfusion and FFR after revascularization. Mean ± SD are displayed. Only revascularized vessels with measurements before and after revascularization were included for this sub analysis. Abbreviations: CAD, coronary artery disease; CFR, coronary flow reserve; FFR, fractional flower reserve; hMBF, hyperemic myocardial blood flow; MI, myocardial infarction; PCI, percutaneous coronary intervention

**Fig. 3:**
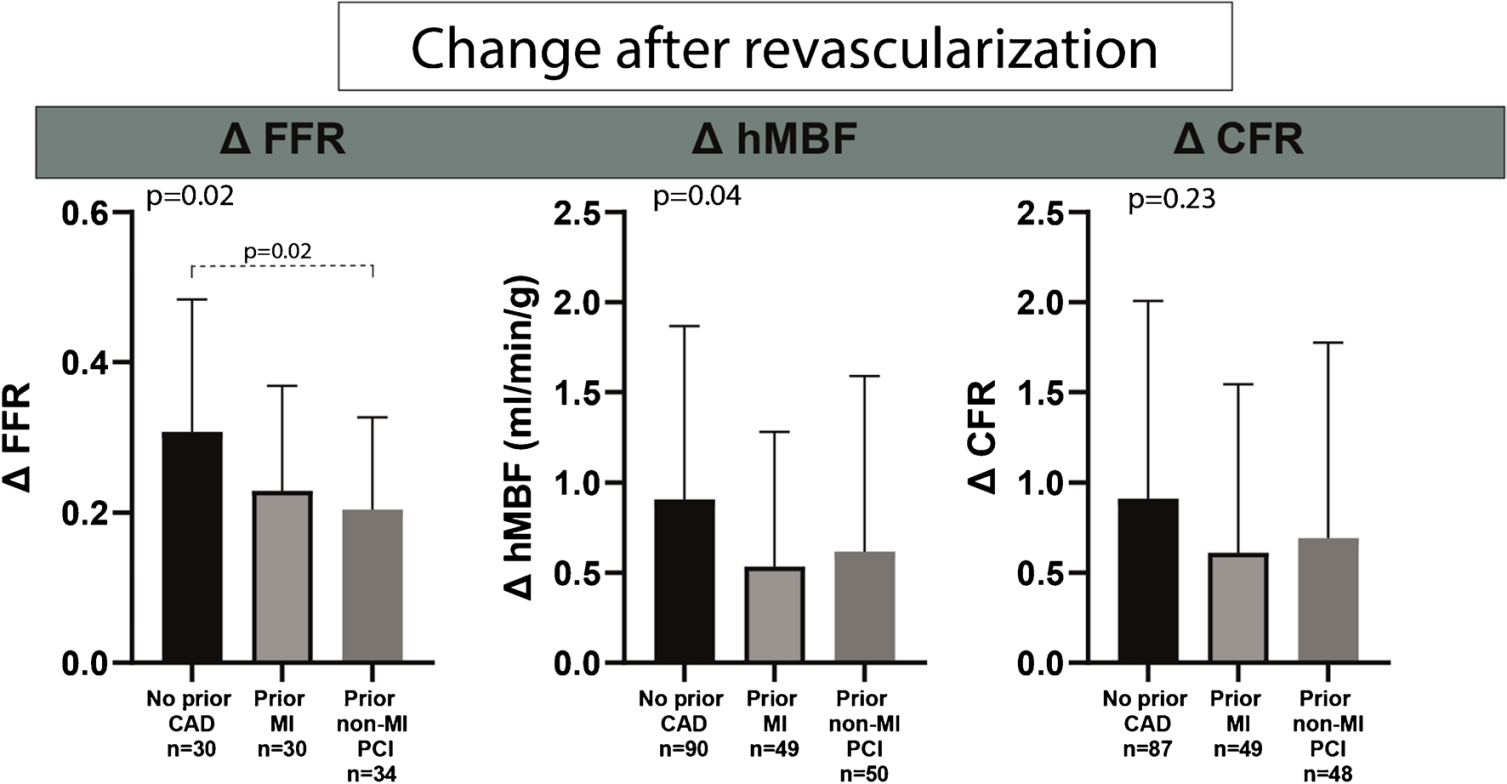
Change in regional perfusion and FFR. Mean ± SD are displayed. Only revascularized vessels with measurements before and after revascularization were included for this sub analysis. Abbreviations: CFR, coronary flow reserve; FFR, fractional flower reserve; hMBF, hyperemic myocardial blood flow

**Table 3:** Perfusion indices and FFR

	Before revascularization	After revascularization	% change	*P* value before—after
Rest MBF				
No prior CAD	0.77 ± 0.16	0.86 ± 025	11.69	< 0.01
Prior MI	090 ± 0.22	0.89 ± 0.21	-1.11	0.88
Prior non-MI PCI	0.91 ± 0.24	0.91 ± 0.22	0.00	0.95
*P* value between groups	< 0.01	0.36		
Hyperemic MBF				
No prior CAD	1.53 ± 0.54	2.44 ± 0.89	59.48	< 0.01
Prior MI	1.86 ± 0.85	2.40 ± 0.88	29.03	< 0.01
Prior non-MI PCI	1.90 ± 0.86	2.55 ± 0.85	34.21	< 0.01
*P* value between groups	< 0.01	0.68		
CFR				
No prior CAD	2.02 ± 0.69	2.92 ± 0.95	44.55	< 0.01
Prior MI	2.11 ± 0.89	2.73 ± 0.98	29.38	< 0.01
Prior non-MI PCI	2.15 ± 0.90	2.85 ± 0.97	32.56	< 0.01
*P* value between groups	0.61	0.49		
FFR				
No prior CAD	0.58 ± 0.19	0.89 ± 0.08	53.44	< 0.01
Prior MI	0.67 ± 0.12	0.90 ± 0.07	34.33	< 0.01
Prior non-MI PCI	0.71 ± 0.10	0.91 ± 0.07	28.17	< 0.01
*P* value between groups	< 0.01	0.39		

Only territories with paired resting MBF and hyperemic MBF before and after revascularization were included in this baseline table

*CAD* coronary artery disease; *MI* myocardial infarction; *PCI* percutaneous coronary intervention

### Change of perfusion and FFR after revascularization

Figure 1 exemplifies the study protocol by showing two case examples and their respective serial PET perfusion scans, ICA and CMR images. After revascularization mean FFR increased from 0.65 ± 0.15 to 0.90 ± 0.08 (*p* < 0.01, Fig. 2). An increase in FFR was observed for patients without a cardiac history, with prior MI or with non-MI PCI. However, it may be appreciated from Fig. 3 that FFR increased to a lesser degree in patients with a prior non-MI PCI compared to those without a prior history (∆ FFR 0.20 ± 0.12 vs. 0.31, *p* = 0.02). Regional hyperemic MBF and CFR improved after revascularization (hMBF: 1.73 ± 0.75 to 2.47 ± 0.88 ml/min/g; CFR: 2.08 ± 0.80 to 2.85 ± 0.96, *p* < 0.01 for both, Fig. 2). Similar to FFR, Fig. 3 shows a trend of an attenuated regional hMBF increase in patients with a prior MI or non-MI PCI (*p* = 0.04, *p* = ns for subgroup differences). CFR increase after revascularization did not significantly differ between patients with or without a prior cardiac history (*p* = 0.23). Baseline FFR and hMBF were significantly lower in patients without prior CAD (*p* < 0.01, Table 3). Post revascularization FFR, hMBF and CFR were similar across all subgroups (*p* = 0.39, *p* = 0.68 and *p* = 0.49). A perfusion decrease after revascularization was seen in 37 territories (ΔhMBF: 0.36 ± 0.39 ml/min/g). Those territories were predominantly characterised by a relatively preserved hMBF at baseline (2.27 ± 0.95 vs 1.60 ± 0.64, *p* < 0.01) and were more prevalent in patients with prior MI or non-MI PCI (27.3% vs 11.1% of revascularized territories, *p* < 0.01). In a sensitivity analysis the absolute improvement in FFR and perfusion indices stratified for baseline FFR (lower or greater than 0.75) are shown in in supplemental Table 1. Patients with a FFR below 0.75 at baseline had a numerically greater FFR and perfusion improvement across all subgroups.

### Relation between FFR and perfusion

**Fig. 4:**
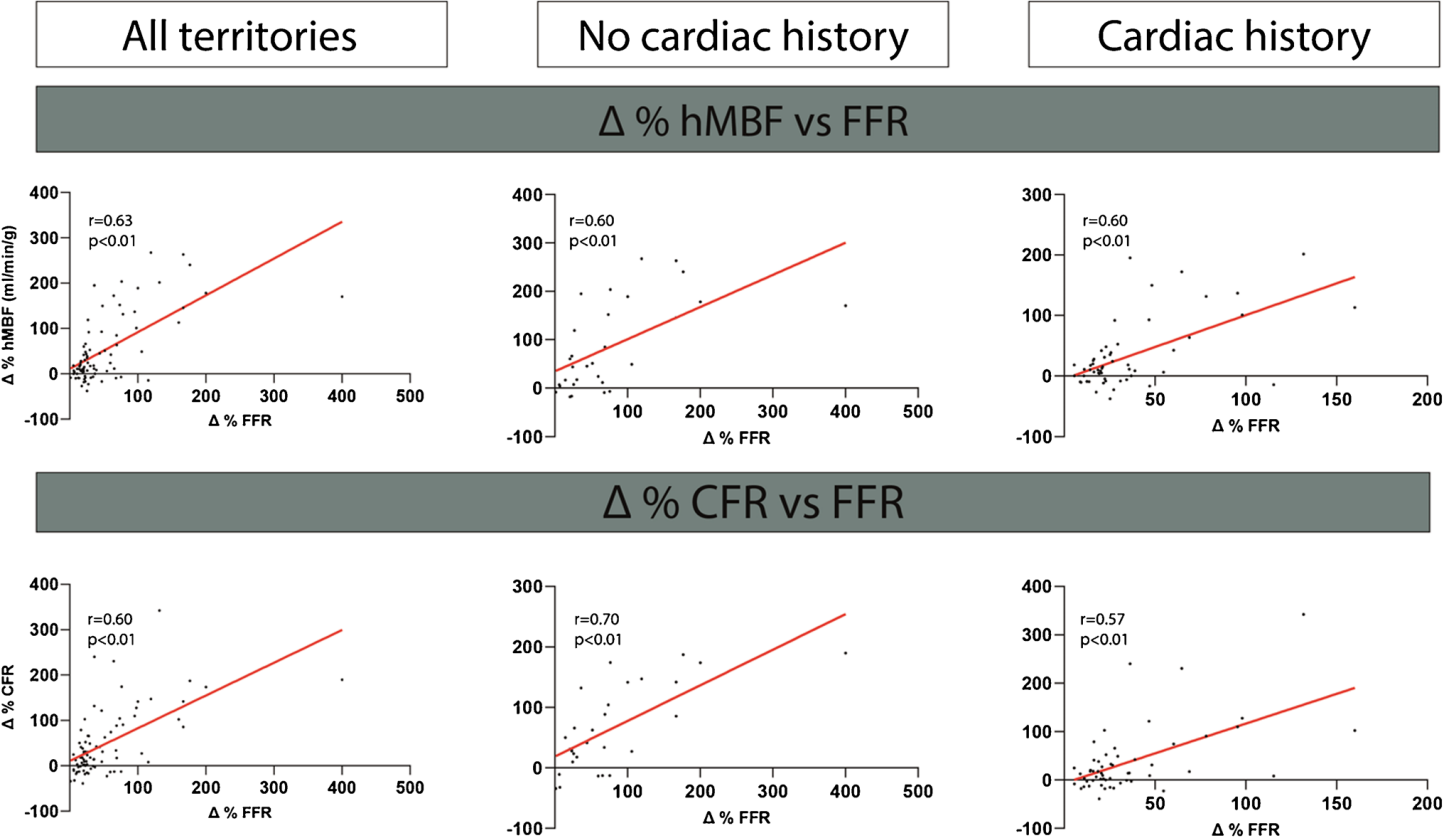
Relationship between changes in FFR and perfusion. Only revascularized vessels with measurements before and after revascularization were included for this sub analysis. A cardiac history was defined as a history of PCI and/or MI. Analyses for patients with a cardiac history could not be split for prior MI/PCI since a limited amount of combined measurements was available. Abbreviations: CFR, coronary flow reserve; FFR, fractional flow reserve; hMBF, hyperemic myocardial blood flow

At baseline, a moderate correlation was observed in patients without a cardiac history between perfusion indices (hMBF and CFR) and FFR (r = 0.59 and r = 0.56, *p* < 0.01 for both). A weak baseline correlation was observed between baseline FFR and perfusion in patients with prior MI or non-MI PCI (hMBF: r = 0.31; CFR r = 0.35 for patients with prior MI and hMBF: r = 0.30; CFR r = 0.22 for patients with prior non-MI PCI, *p* < 0.01 for all, supplemental Fig. 4). Hyperemic MBF and FFR were at baseline concordant in 72% of patients without cardiac history, whereas 58% and 57% of the measurements were concordant in patients with prior MI or non-MI PCI. Figure 4 shows the relation between % perfusion change and FFR following revascularization. The relationship between FFR and hMBF increase was strong in both patients with and without cardiac history (r = 0.60 and r = 0.60, *p* < 0.01 for both). The relationship between FFR and CFR was strong in patients without a cardiac history (r = 0.70, *p* < 0.01) and moderate (r = 0.57, *p* < 0.01) for patients with a cardiac history. A substantial overlap was observed between the segment involvement scores among patients without prior CAD, with prior MI or with prior non-MI PCI with more segments affected by atherosclerotic disease in patients without prior CAD than in patients with prior MI or non-MI PCI (supplemental Table 2).

### The influence of scar on change of FFR and perfusion

**Fig. 5:**
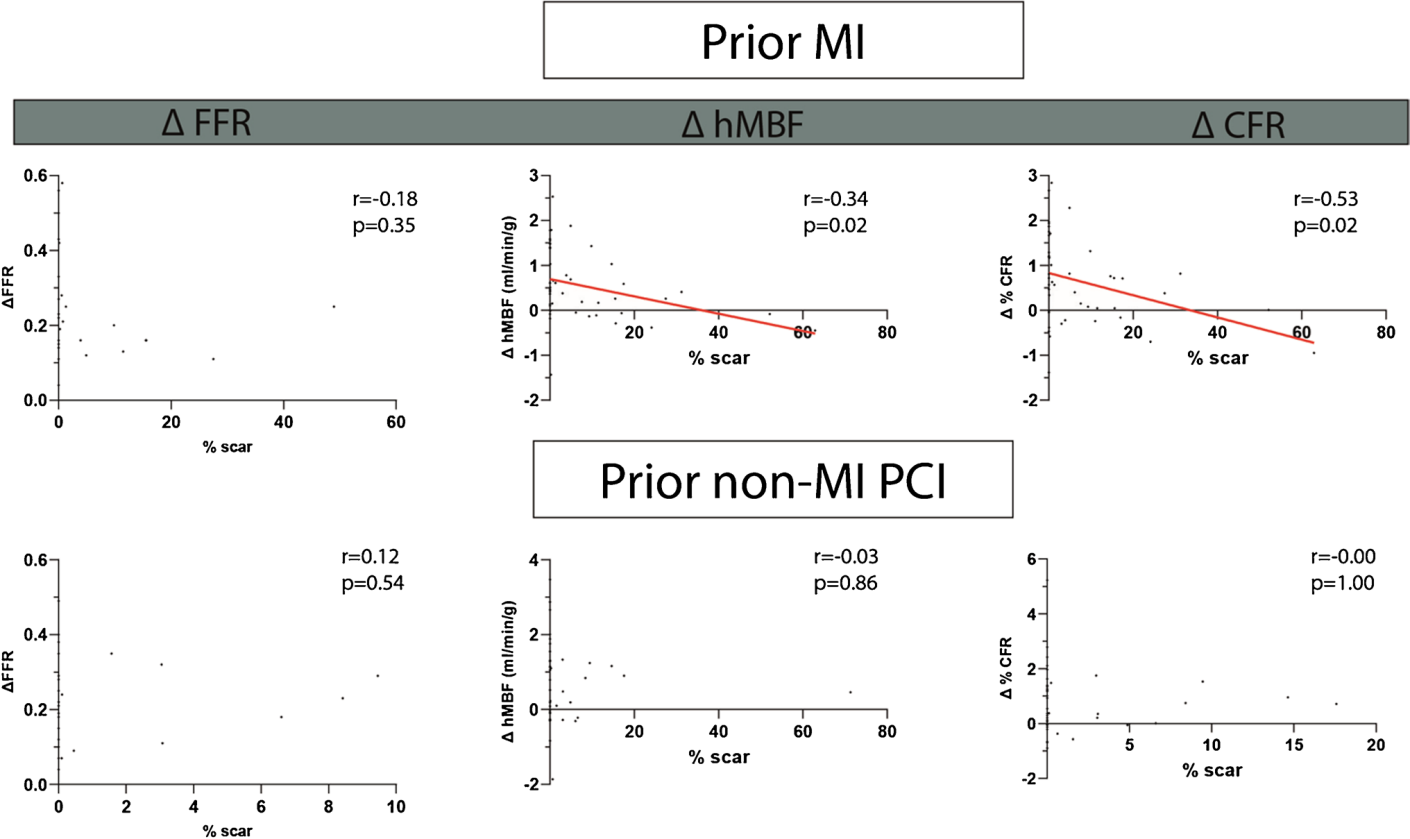
Relationship between scar and change in FFR and perfusion. The relation between scar and delta FFR (*n* = 57), delta hMBF (*n* = 94) and CFR (*n* = 92) split for patients with prior MI and prior non-MI PCI. Scar is depicted as a percentage of the revascularized segments. Abbreviations: CFR, coronary flow reserve; FFR, fractional flow reserve; hMBF, hyperemic myocardial blood flow; MI, myocardial infarction; PCI, percutaneous coronary intervention

Table 2 depicts the amount of LGE per revascularized territory. Figure 5 shows the impact of regional scar tissue on restoration of regional myocardial perfusion. Increased scar was negatively associated to hMBF and CFR increase (r = -0.34, *p* = 0.02 and r = -0.53, *p* = 0.02) in patients with prior MI. In patients with non-MI PCI scar and perfusion were not related.

### Predictors of perfusion improvement

In a univariable analysis (supplemental Table 3) including baseline characteristics, cardiovascular risk factors, prior PCI for stable CAD in the revascularized territory, left ventricle ejection fraction and scar (as % of revascularized territory) only prior PCI for stable CAD in the revascularized territory and scar had a *p* < 0.15 for hMBF increase. Scar and a history of smoking were negative predictors of CFR increase. In the multivariable analysis assessing hMBF increase, none of the predictors with a univariable p-value < 0.15 were significantly associated with hMBF increase. Scar and a history of smoking were independent negative predictors of CFR increase.

## Discussion

This sub study of the PACIFIC 1 and 2 assessed the potential of coronary revascularization to restore myocardial perfusion as assessed by quantitative [^15^O]H_2_O PET and is, to the best of our knowledge, the first to evaluate the effect of prior coronary revascularizations and myocardial infarctions on the improvement of absolute myocardial perfusion after revascularization. Moreover, this study provides insight into the relationship between FFR and absolute myocardial perfusion before and after revascularization. The main findings can be summarized as follows: 1) Successful coronary revascularization improved FFR and perfusion in patients without a cardiac history, with prior MI or with prior non-MI PCI 2) Post revascularization FFR and perfusion were similar in patients without a cardiac history, with prior MI or non-MI PCI 3) Changes in FFR and absolute perfusion were strongly associated 4) Regional scar and a history of smoking were independently negatively associated with CFR increase. Other cardiovascular risk factors were not independently predictive of an attenuated recovery of myocardial perfusion.

### Functional treatment of CAD

Physiology-guided coronary interventions have been shown to confer prognostic benefit over a merely anatomical driven approach [13]. Germane to this, post-PCI FFR contains prognostic information and a suboptimal restoration of intracoronary pressures (i.e. FFR < 0.90) has been associated with an increased risk of adverse cardiovascular events and higher rates of target vessel revascularization [14, 15]. Therefore, FFR has been considered the reference standard for discerning the functional significance of epicardial lesions [1]. However, the question remains whether FFR and myocardial perfusion metrics are interchangeable and provide us with similar or complementary information on the CAD spectrum. Interestingly, FFR and [^15^O]H_2_O PET have a shared history. The second ever published paper on FFR was its first validation in humans in isolated LAD lesions using relative flow reserve by [^15^O]H_2_O PET as a reference standard [16]. Several studies confirmed that FFR provides an invasive measure of myocardial perfusion, although the relationship is governed by diffuse epicardial disease, and microvascular function. Contrary to the validation study of the de Bruyne et al. we included symptomatic patients and did not exclude diffusely diseased patients, which is exemplified by median segment involvement scores ≥ 4. In this regard, we found a moderate correlation between baseline FFR, hyperemic MBF and CFR in patients without a prior cardiac history, which is consistent with earlier results [17]. On the other hand, only a weak correlation was seen between baseline FFR and perfusion metrics in patients with prior MI or non-MI PCI. Patients with prior CAD had lower segment involvement scores suggesting more diffuse CAD in patients without prior CAD. The weaker baseline correlation between FFR and perfusion in patients with prior CAD may be ascribed to a higher occurrence of microvascular disease in this high-risk category. Also, the influence of plaque characteristics on FFR and perfusion cannot be neglected [18, 19]. Patients with prior CAD (I.e. prior MI or non-MI PCI) had a numerically lower FFR and perfusion increase. It should be noted that baseline perfusion and FFR of patients without prior CAD were lower but post revascularization FFR and perfusion were similar, suggesting a similar post revascularization potential. Therefore, non-invasive evaluation of myocardial perfusion by PET may play a role in identifying patients who might experience symptom reduction from revascularization therapy. Quantitative myocardial blood flow imaging is nowadays not only limited to PET MPI. There are promising results using CZT-SPECT devices. Although there are important differences in tracer kinetics, it is promising Acampa and colleagues have demonstrated the feasibility of quantifying myocardial perfusion using CZT-SPECT [20]. Furthermore, Mannarino et al. showed that vessels with > 50% diameter stenosis on invasive angiography with regional perfusions deficits showed a trend towards more vessel-specific adverse events, underlying the potential of functional imaging to guide coronary interventions and to predict revascularization related outcome [21]. Integrating anatomical and functional information offers several advantages, while independently used may lead to disregarding valuable information on important aspects of the complex interplay between anatomy and ischemia.

Notably, multiple perfusion metrics can be derived from PET. A study by Bom and colleagues demonstrated longitudinal perfusion gradients to reflect diffuse CAD, while Johnson and colleagues suggested the integration of hMBF and CFR into coronary flow capacity to more accurately identify ischemia [22]. A serial PET study by De Winter et al. revealed coronary flow at baseline to predict improvement of perfusion after revascularization therapy [23, 24]. Most probably the abovementioned perfusion metrics are complementary and should all be evaluated in symptomatic patients [25]. Further studies need to exploit these perfusion metrics to advance our understanding of the atherosclerotic spectrum and on the potential future applications of quantitative PET beyond its current role being merely a gatekeeper for the “cathlab”.

### The influence of scar and risk factors on restoration of perfusion

Restoration of myocardial perfusion following revascularization is dependent on the alleviation of coronary diameter stenosis. However, myocardial blood flow is regulated by both epicardial coronary flow and microvascular resistance. Traditional risk factors have been shown to induce microvascular disease, a condition that is associated with abnormal myocardial perfusion requiring specific conservative (i.e. noninvasive) treatment [26–28]. Although microvascular disease represents a separate entity with unique therapeutic implications, there is a large overlap between epicardial atherosclerosis and microvascular dysfunction [29]. Residual ischemia after successful coronary revascularization could be caused by residual CAD or microvascular dysfunction. We assessed the diffuseness of atherosclerosis by calculating visual segment involvement scores. Prior studies suggested that diffuse, potentially flow-limiting, CAD could be missed by visual assessment [30]. These patients are at risk of being incorrectly classified as patients with microvascular disease, potentially attributing to a significant proportion of contemporary patients diagnosed with microvascular disease [29].

The amount of ischemia that is accounted for by diffuse CAD or microvascular disease is difficult to determine upfront and has been associated with persisting angina and even adverse outcomes. In other words, a successful stent placement is not necessarily commensurate with improved perfusion. Additionally, in a study from our institution, lower stress perfusion was seen in elderly and obese patients, despite focal obstructive CAD was excluded [31].

In the present study, of conventional risk factors only a history of smoking was predictive of perfusion increase. This may be attributed to a population with a relatively large atherosclerotic burden, an end-stage disease wherein the impact of traditional risk factors may be nullified. The vascular damage caused by traditional cardiovascular risk factors attenuates the relative contribution of these risk factors. Indeed, studies have shown that prediction models perform better in middle-aged populations than in elderly patients [32]. In addition, risk scores appear to have only a modest discriminatory power in high-risk patients with typical angina [33]. The presence and extent of regional scar in patients with a prior MI was associated to an attenuated perfusion increase and independently predictive of a attenuated CFR increase following revascularization. We found slightly stronger correlations between scar and CFR than between scar and hMBF. One explanation is that CFR incorporates rest perfusion. An increased rest perfusion is associated to MACE and related to microvascular dysfunction and epicardial coronary stenosis [34]. It bears mentioning that scar burden in our population was rather small and FFR measurements account for scarred myocardium. Territories with extensive scar will by definition have a higher FFR and will be less likely revascularized [35]. In addition, the use of a [^15^O]H_2_O perfusion tracer is less suitable for visualizing non-viable tissue since MBF is only measured in viable tissue and this may have affected the present findings. Nevertheless, scar tissue is often heterogeneous and contains islands of viable tissue, which is reflected by [^15^O]H_2_O PET as areas of abnormal perfusion. Interestingly, a recent animal study by Grönman and colleagues demonstrated resting MBF suitable for the assessment of viability with a similar diagnostic value as measures of perfusable tissue fractions and index [36]. All-in all, epicardial atherosclerosis and coronary microvascular dysfunction are two different entities of the atherosclerotic spectrum with large overlap. Non-invasive quantitative perfusion imaging combined with FFR measurements may provide a comprehensive understanding of the complex interplay between FFR, microvascular function and its impact on myocardial perfusion. We studied the influence of revascularization on perfusion and FFR restoration but had no information on the post revascularization symptomatic status. As such we could not correlate our findings with (post)revascularization symptoms. Also the numbers do not allow a prognostic analysis. However, since ischaemia testing and FFR are the backbone of the revascularization strategy we think these derivatives are of sufficient quality to provide useful information. Further studies are warranted to determine whether a combined approach of pressure and flow measures may further distinguish between patients who may benefit from revascularization and patients in whom pharmacotherapy targeting endothelial and microvascular atherosclerosis is required in terms of perfusion increase and symptom reduction.

### Limitations

This is a post hoc sub analysis of two prospective studies including a modest number of patients, and some limitations should be addressed. First, although coronary dominance was checked regional perfusion as assessed by PET is matched with FFR measurements upon standardized coronary anatomy and does not account for individual variations. Second, [15^O^]H_2_O was used as tracer. The tracer has the unique ability to be linearly related to myocardial blood flow, primarily in viable myocardium [37]. Therefore the influence of nonviable tissue (i.e. scar) could be underestimated. Third, although patients with prior MI were included, regional extensive scar was scarce and results with regard to the influence of scar should be considered hypothesis-generating. This might be attributed to the excellent STEMI care in the densely populated Netherlands [38]. Fourth, segment involve scores were calculated to analyze diffuseness of atherosclerosis. Pressure pull back curves were not routinely performed during ICA. Finally, patients underwent follow-up PET after a median of 34 days. It has been reported that myocardial perfusion increases further after 1 month post revascularization. Therefore myocardial perfusion might have increased further in our patients [39, 40].

## Conclusion

Successful coronary revascularization improved FFR and absolute myocardial perfusion in patients without a cardiac history, with prior MI or with prior non-MI PCI. Post revascularization FFR and perfusion were similar in patients without a cardiac history, with prior MI or non-MI PCI. An increase in FFR was paralleled by improvements in absolute perfusion. In patients with prior infarction scar burden was associated to an attenuated perfusion increase.

### Supplementary Information

Below is the link to the electronic supplementary material.

Supplementary file1 (DOCX 2.59 kb)

## Data Availability

Data are available on request.

## Abbreviations

CABG: Coronary artery bypass graft surgery
CAD: Coronary artery disease
CFR: Coronary flow reserve
CMD: Coronary microvascular dysfunction
FFR: Fractional flow reserve
ICA: Invasive coronary angiography
LGE: Late gadolinium enhancement
hMBF: Hyperemic myocardial blood flow
MI: Myocardial infarction
CMR: Cardiac magnetic resonance
PCI: Percutaneous coronary intervention
PET: Positron emission tomography
