# Supplementary material for: Impact of cardiac history and myocardial scar on increase of myocardial perfusion after revascularization
Source: Eur J Nucl Med Mol Imaging. 2023 Aug 10;50(13):3897–909. doi: 10.1007/s00259-023-06356-4 (PMC10611874; doi:10.1007/s00259-023-06356-4)
Supplement: Supplementary file 1 — Supplementary file1 (DOCX 2.59 kb) [file 259_2023_6356_MOESM1_ESM.docx]

**Supplemental table 1. Analysis stratified for FFR≥0.75 or <0.75.**

|  | Before revascularization | After revascularization | Change | P value  before - after |  |
| --- | --- | --- | --- | --- | --- |
| **FFR (FFR <0.75)** | | | | | |
| No prior CAD (n=22) | 0.50 ± 0.16 | 0.87 ± 0.08 | 0.37 ± 0.16 | <0.01 |  |
| Prior MI (n=23) | 0.64 ± 0.12 | 0.89 ± 0.07 | 0.25 ± 0.15 | <0.01 |  |
| Prior non-MI PCI (n=21) | 0.66 ± 0.09 | 0.92 ± 0.08 | 0.25 ± 0.12 | <0.01 |  |
| **FFR (FFR ≥0.75)** | | | | | |
| No prior CAD (n=8) | 0.80 ± 0.03 | 0.93 ± 0.06 | 0.14 ± 0.07 | <0.01 |  |
| Prior MI (n=7) | 0.78 ± 0.04 | 0.94 ± 0.06 | 0.16 ± 0.04 | <0.01 |  |
| Prior non-MI PCI (n=13) | 0.79 ± 0.04 | 0.91 ± 0.07 | 0.12 ± 0.07 | <0.01 |  |
| **Hyperemic MBF (FFR <0.75)** | | | | | |
| No prior CAD (n=45) | 1.63 ± 0.45 | 2.73 ± 1.05 | 1.10 ± 1.09 | <0.01 |  |
| Prior MI (n-24) | 2.07 ± 0.82 | 2.70 ± 0.68 | 0.63 ± 0.72 | <0.01 |  |
| Prior non-MI PCI (n=25) | 1.81 ± 0.73 | 2.24 ± 0.85 | 0.44 ± 0.98 | 0.04 |  |
| **Hyperemic MBF (FFR ≥ 0.75)** | | | | | |
| No prior CAD (n=16) | 2.23 ± 0.65 | 2.43 ± 0.66 | 0.20 ± 0.82 | 0.25 |  |
| Prior MI (n=13) | 2.22 ± 0.80 | 2.56 ± 0.88 | 0.34 ± 0.80 | 0.15 |  |
| Prior non-MI PCI (13) | 2.42 ± 0.92 | 2.81 ± 0.91 | 0.39 ± 0.97 | 0.18 |  |
| **CFR (FFR <0.75)** | | | | | |
| No prior CAD (n=43) | 2.13 ± 0.71 | 3.11 ± 0.79 | 0.98 ± 1.08 | <0.01 |  |
| Prior MI (n=24) | 2.25 ± 0.90 | 3.03 ± 0.74 | 0.78 ± 0.87 | <0.01 |  |
| Prior non-MI PCI (n=24) | 2.10 ± 0.80 | 2.71 ± 1.16 | 0.62 ± 1.17 | 0.02 |  |
| **CFR (FFR ≥0.75)** | | | | | |
| No prior CAD (n=15) | 2.63 ± 0.52 | 2.80 ± 1.03 | 0.17 ± 0.82 | 0.44 |  |
| Prior MI (n=13) | 2.59 ± 0.77 | 2.88 ± 1.17 | 0.30 ± 1.06 | 0.34 |  |
| Prior non-MI PCI (12) | 2.58 ± 1.09 | 2.92 ± 0.71 | 0.34 ± 0.80 | 0.17 |  |
| **Delta baseline FFR <0.75 vs FFR ≥0.75** | | | | | |
|  | Delta <0.75 | Delta FFR ≥ 0.75 |  |  |  |
| FFR |  |  |  |  |  |
| No prior CAD | 0.37 ± 0.16 | 0.14 ± 0.07 |  | <0.01 |  |
| Prior MI | 0.25 ± 0.15 | 0.16 ± 0.04 |  | <0.01 |  |
| Prior non-MI PCI | 0.25 ± 0.12 | 0.12 ± 0.07 |  | <0.01 |  |
| hMBF |  |  |  |  |  |
| No prior CAD | 1.10 ± 1.09 | 0.20 ± 0.82 |  | <0.01 |  |
| Prior MI | 0.63 ± 0.72 | 0.34 ± 0.80 |  | 0.66 |  |
| Prior non-MI PCI | 0.44 ± 0.98 | 0.39 ± 0.97 |  | 0.59 |  |
| CFR |  |  |  |  |  |
| No prior CAD | 0.98 ± 1.08 | 0.17 ± 0.82 |  | 0.01 |  |
| Prior MI | 0.78 ± 0.87 | 0.30 ± 1.06 |  | 0.15 |  |
| Prior non-MI PCI | 0.62 ± 1.17 | 0.34 ± 0.80 |  | 0.47 |  |

Mean ± SD are displayed. Only revascularized vessels with measurements before and after revascularization were included for this sub analysis. Abbreviations: CAD, coronary artery disease; CFR, coronary flow reserve; FFR, fractional flower reserve; hMBF, hyperemic myocardial blood flow; MI, myocardial infarction; PCI, percutaneous coronary intervention

**Supplemental table 2. Segment involvement scores**

| Patient group | No cardiac history (n=53) | Prior MI (n=41) | Prior non-MI PCI (n=43) |
| --- | --- | --- | --- |
| All patients | 8.0 (5.0 -10.0) | 5.0 (3.5 -7.0) | 4.0 (3.0 -6.0) |
| Paired FFR | 6.0 (3.8 -9.5) | 5.0 (3.3 -6.0) | 4.0 (3.0 -6.0) |
| Paired hMBF | 8.0 (5.0 – 10.0) | 5.0 (3.5-6.5) | 4.0 (3.0 -5.3) |
| Paired CFR | 8.0 (5.0 – 10.0) | 5.0 (3.5 – 6.5) | 4.0 (3.0 -5.3) |

Segment involvement scores based on invasive angiography.

Abbreviations: CAD, coronary artery disease; CFR, coronary flow reserve; FFR, fractional flower reserve; hMBF, hyperemic myocardial blood flow; MI, myocardial infarction; PCI, percutaneous coronary intervention

**Supplemental table 3. Linear Mixed Model Identifying predictors of perfusion improvement**

|  | hMBF | | | | CFR | | | |
| --- | --- | --- | --- | --- | --- | --- | --- | --- |
|  | Univariable Analysis | | Multivariable Analysis | | Univariable Analysis | | Multivariable Analysis | |
|  | Beta | P value | Beta | P value | Beta | P value | Beta | P value |
| Patient characteristics |  | | | | | | | |
| Age (per 10 y) | 0.00 | 0.72 |  | n/a | 0.00 | 0.71 |  | n/a |
| Male sex | -0.43 | 0.17 |  | n/a | -0.02 | 0.96 |  | n/a |
| BMI (per kg/m2 increase) | 0.00 | 0.90 |  | n/a | -0.01 | 0.63 |  | n/a |
| Diabetes Mellitus | -0.06 | 0.78 |  | n/a | -0.10 | 0.68 |  | n/a |
| Hypertension | 0.02 | 0.92 |  | n/a | 0.06 | 0.81 |  | n/a |
| Hypercholesterolemia | 0.18 | 0.44 |  | n/a | 0.11 | 0.68 |  | n/a |
| History of smoking | -0.22 | 0.24 |  | n/a | -0.41 | 0.06 | -0.48 | 0.03 |
| Family history of CAD | 0.22 | 0.24 |  | n/a | 0.04 | 0.86 |  | n/a |
| Prior PCI for stable CAD in revascularized territory | 0.29 | 0.11 | 0.36 | 0.06 | 0.25 | 0.27 |  | n/a |
| Scar (per % of revascularized territory) | -0.01 | 0.13 | -0.01 | 0.16 | -0.02 | 0.04 | -0.02 | 0.04 |
| Ejection fraction (%) | 0.01 | 0.18 |  |  | 0.01 | 0.41 |  | n/a |

Variables were included in the multivariable analysis if univariably related to perfusion improvement (p<0.15). Abbreviations: BMI, body mass index; CAD, coronary artery disease; CFR, coronary flow reserve; hMBF, hyperemic myocardial blood flow; PCI, percutaneous coronary intervention


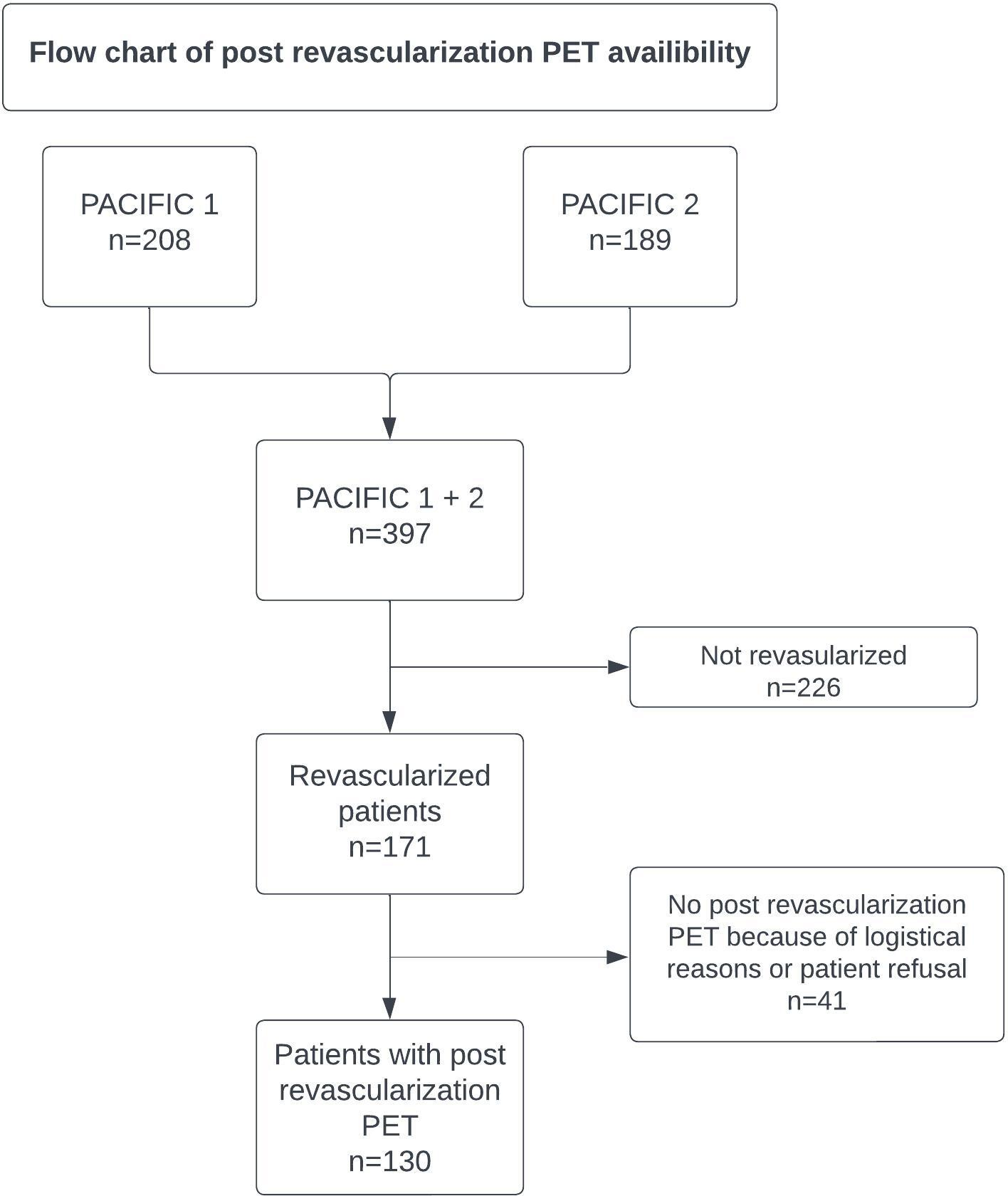


**Supplemental figure 1. Flowchart depicting PET availability**

Abbreviations: PET, positron emission tomography


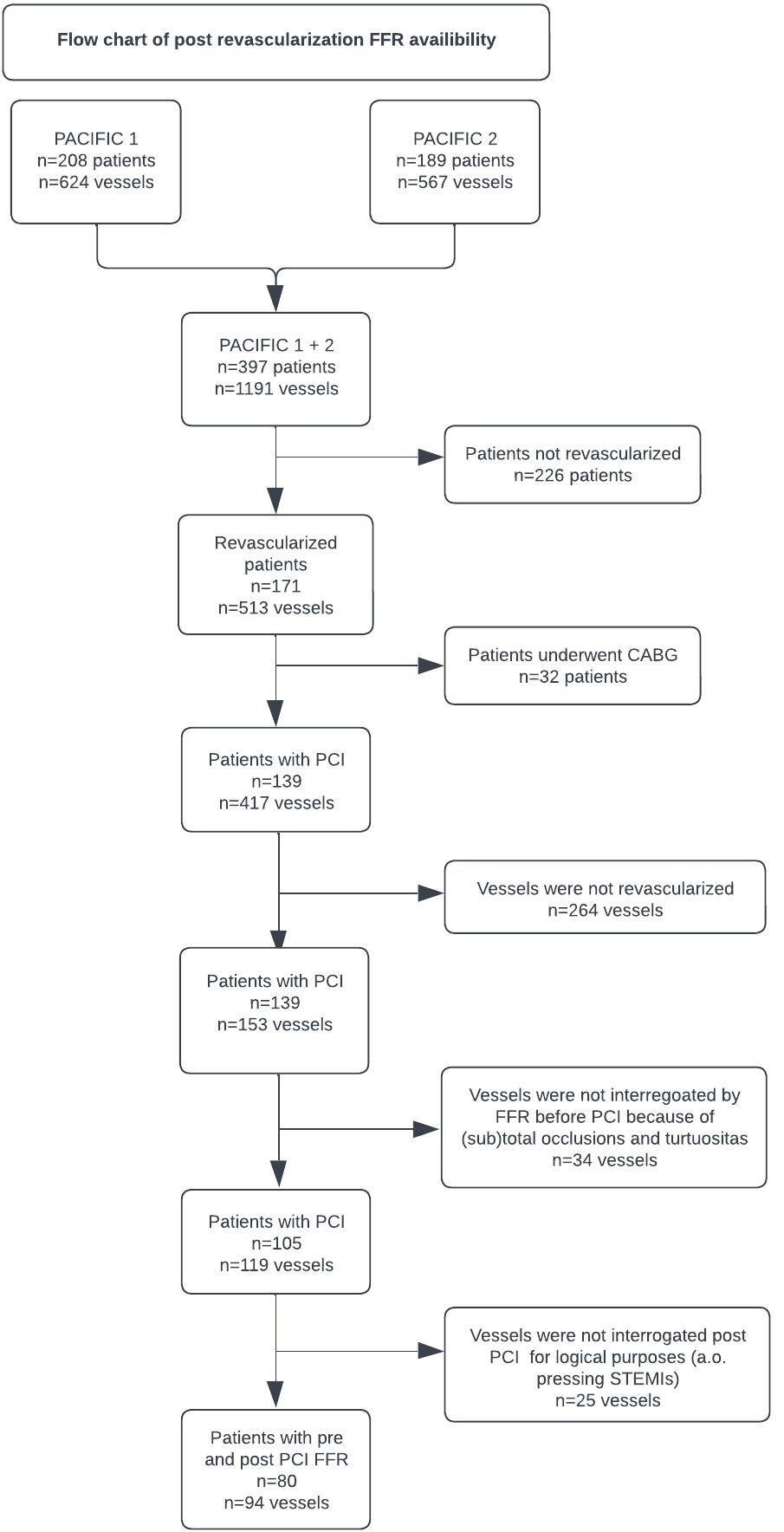


**Supplemental figure 2. Flowchart depicting post PCI FFR availability**

Abbreviations: CABG, coronary artery bypass grafting; FFR, fractional flow reserve; PCI, percutaneous coronary intervention;


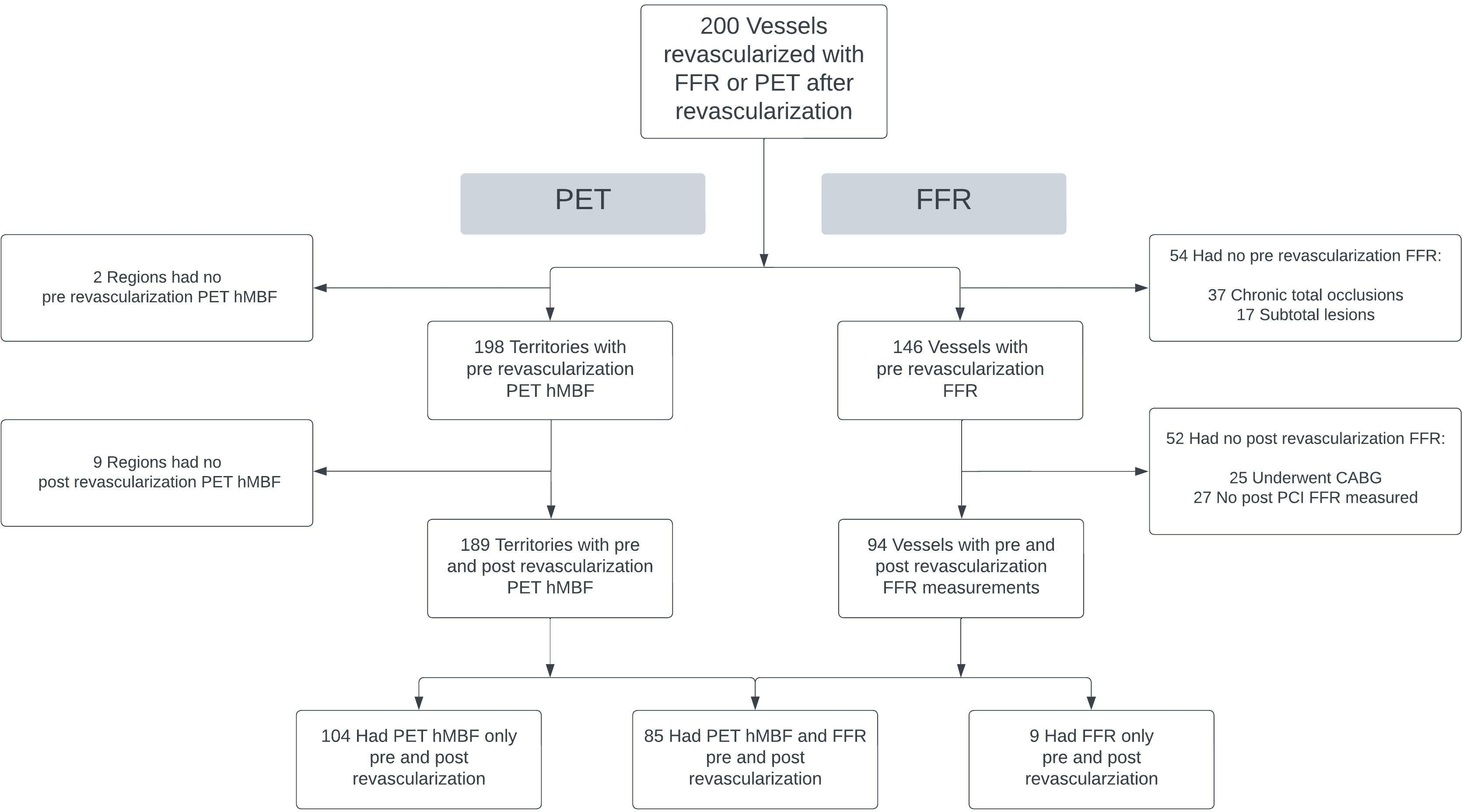


**Supplemental figure 3. Flowchart depicting FFR and hMBF availability per vessel/territory.**

Abbreviations: FFR, fractional flower reserve; hMBF, hyperemic myocardial blood flow; PET, positron emission tomography


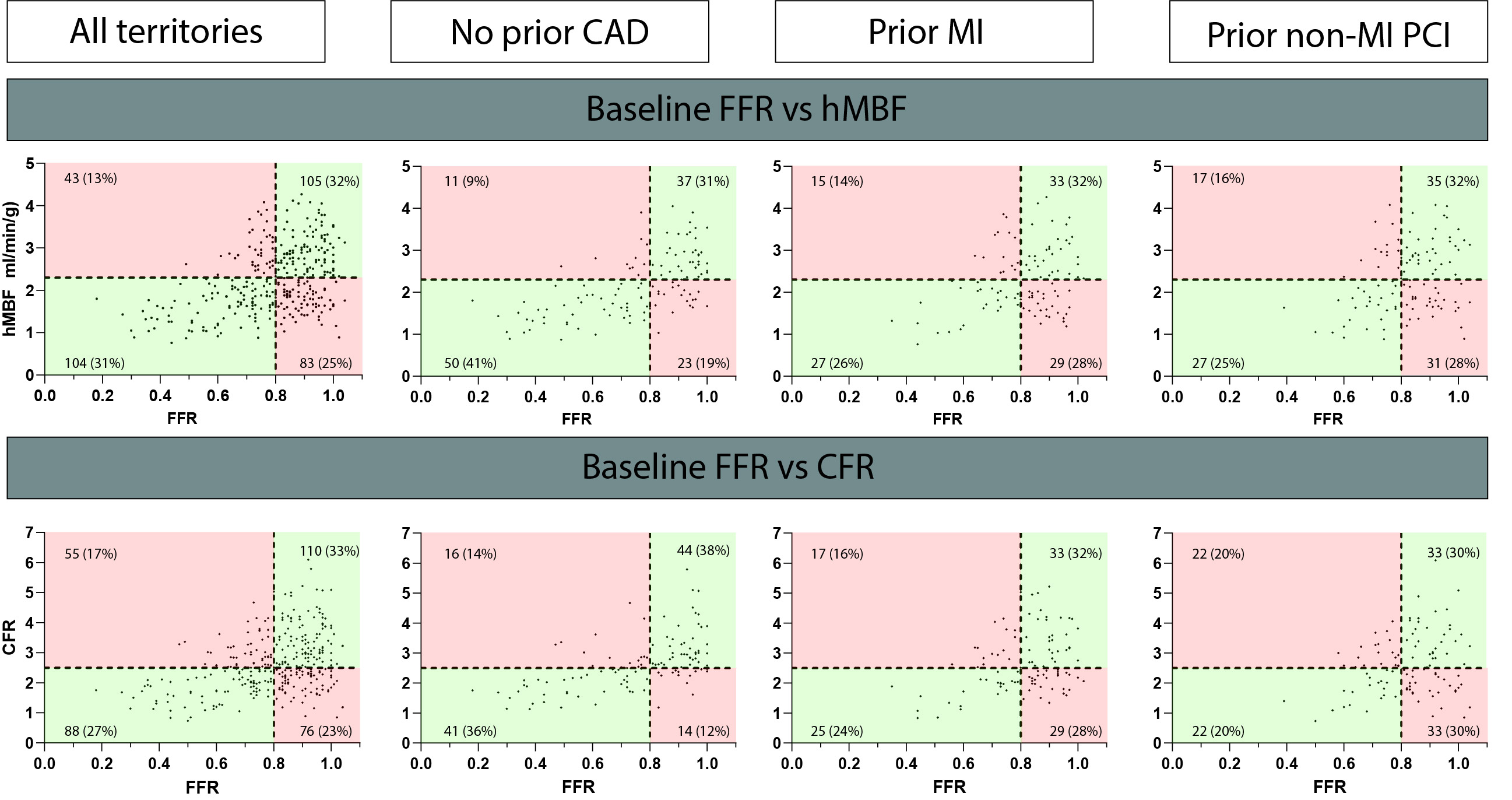


**Supplemental figure 4. Relationship between regional perfusion and FFR at baseline**

Abbreviations: CFR, coronary flow reserve; FFR, fractional flower reserve; hMBF, hyperemic myocardial blood flow; MI, myocardial infarction; PCI, percutaneous coronary intervention
